# Adhesion pathway proteins and risk of atrial fibrillation in the Multi-Ethnic Study of Atherosclerosis

**DOI:** 10.1186/s12872-021-02241-w

**Published:** 2021-09-14

**Authors:** Israel J. Mendez, Sheila M. Manemann, Elizabeth J. Bell, Nicholas B. Larson, Paul A. Decker, Marco A. Guerrero, Naomi Q. Hanson, Susan R. Heckbert, James S. Pankow, Michael Y. Tsai, Suzette J. Bielinski

**Affiliations:** 1 Department of Quantitative Health Sciences, Mayo Clinic, 200 First Street Southwest, Rochester, MN USA; 2 Department of Cardiovascular Diseases, Mayo Clinic, Rochester, MN USA; 3 Regions Hospital-The Heart Center, St. Paul, MN USA; 4 Department of Laboratory Medicine and Pathology, School of Medicine, University of Minnesota, Minneapolis, MN USA; 5 Cardiovascular Health Research Unit and Department of Epidemiology, University of Washington, Seattle, WA USA; 6 Division of Epidemiology and Community Health, School of Public Health, University of Minnesota, Minneapolis, MN USA; 7 Present Address: University of Puerto Rico-School of Medicine, San Juan, PR USA; 8 Present Address: Optum, Life Sciences, Eden Prairie, MN USA

**Keywords:** Adhesion molecules, Atrial fibrillation, Inflammation, Risk factors

## Abstract

**Background:**

The cellular adhesion pathway has been suggested as playing an important role in the pathogenesis of atrial fibrillation (AF). However, prior studies that have investigated the role of adhesion pathway proteins in risk of AF have been limited in the number of proteins that were studied and in the ethnic and racial diversity of the study population. Therefore we aimed to study the associations of fifteen adhesion pathway proteins with incident AF in a large, diverse population.

**Methods:**

Multi-Ethnic Study of Atherosclerosis participants from four races/ethnicities (n = 2504) with protein levels measured were followed for incident AF (n = 253). HGF protein was measured on Exam 1 samples (N = 6669; AF n = 851). Cox proportional hazards regression was used to assess the association of AF with 15 adhesion pathway proteins. Bonferroni correction was applied to account for multiple comparisons.

**Results:**

After adjusting for potential confounding variables (age, sex, race/ethnicity, height, body mass index, systolic blood pressure, antihypertension therapy, diabetes status, current smoker, current alcohol use, and total and HDL cholesterol), and accounting for multiple testing (*P* < 0.05/15 = 0.0033), circulating levels of the following proteins were positively associated with a higher risk of AF: MMP-2 (HR per standard deviation increment, 1.27; 95% CI 1.11‒1.45), TIMP-2 (HR 1.28; 95% CI 1.12‒1.46), VCAM-1 (HR 1.32; 95% CI 1.16‒1.50), and SLPI (HR 1.22; 95% CI 1.07‒1.38). The association between proteins and AF did not differ by race/ethnicity.

**Conclusions:**

Circulating levels of MMP-2, TIMP-2, VCAM-1, and SLPI were positively associated with an increased risk of incident AF in a diverse population. Our findings suggest that adhesion pathway proteins may be important risk predictors of AF.

**Supplementary Information:**

The online version contains supplementary material available at 10.1186/s12872-021-02241-w.

## Background

Atrial fibrillation (AF) is the most common chronic cardiac arrhythmia, with an estimated prevalence in 2010 of between 3 and 6 million individuals in the United States [1, 2]. Although widely studied, the pathophysiology of AF is not completely understood. Inflammation has been suggested as an underlying mechanism and a risk factor for AF [3–5].

The Framingham Offspring Study assessed the association of 12 circulating inflammatory markers and found that circulating osteoprotegerin was associated with incident AF [6].Furthermore, there is growing evidence that the cellular adhesion pathway, a biological pathway of inflammation, may play a role in AF. For example, in the Women’s Health Study, markers of systemic inflammation, including high sensitivity C-reactive protein (CRP), soluble intercellular adhesion molecule-1 (ICAM-1), and fibrinogen, were significantly associated with AF [7]. A prospective, population-based cohort study that studied thirteen inflammation markers found that the vascular cell adhesion molecule 1 (VCAM-1) was significantly associated with new-onset AF in the general community [8].

However, there are important gaps in our knowledge of the relationship between adhesion pathway proteins and risk of AF. Prior studies included only a limited number of adhesion pathway proteins and have been limited in their ethnic/racial diversity [6–10]. Thus, the aim of this study was to determine the association of fifteen adhesion pathway proteins with risk of AF in the diverse Multi-Ethnic Study of Atherosclerosis (MESA).

## Methods

### Study population

The MESA study, as previously described, recruited 6,814 African, Chinese, Hispanic, and non-Hispanic white Americans aged 45 to 84 years (47% male) with no history of cardiovascular disease (CVD) [11]. Hepatocyte growth factor (HGF) was measured at Exam 1 in 6669 participants. A stratified random sample including 720 individuals for each of the 4 races/ethnicities (n = 2880) was used to measure the circulating levels of adhesion pathway proteins at Exam 2. There were 2550 subjects with plasma and/or serum and AF data available. Of these, 30 had a CVD event prior to Exam 2 and were excluded. An additional 16 had an AF event prior to Exam 2 and were excluded, thus resulting in 2504 subjects for analysis (see Additional file 1: Figure S1). All the participants gave written informed consent, and the MESA study protocol and its ancillary studies received approval by the appropriate ethics committees: the University of Minnesota Institutional Review Board (IRB) and the IRBs at all of the other participating centers. The study was done in accordance with the Declaration of Helsinki.

### Protein measurements

Blood was obtained from fasting participants at Exam 1 (2000–2002) and Exam 2 (2002–2004) and stored at − 70 °C. Quantitative sandwich enzyme-linked immunosorbent assays were used to measure the adhesion pathway proteins (in years 2010–2012). The 15 circulating proteins were chosen based on biological plausibility of associating with heart disease as determined by published literature or network interaction and had a high-quality commercial assay available. P-selectin, Regulated on Activation, Normal T cell Expressed and Secreted (RANTES), stromal-derived factor 1α (SDF-1α) and transforming growth factor β1 (TGF-β1) were measured in EDTA plasma, while matrix metalloproteinase 1 (MMP-1), matrix metalloproteinase 2 (MMP-2), tissue inhibitor of metalloproteinase 2 (TIMP-2), ICAM-1, VCAM-1, L-selectin, HGF, chemokine ligand 21 (CCL-21), secretory leukocyte protease inhibitor (SLPI), E-cadherin, and interleukin 2 soluble receptor (IL-2 sR) were measured in serum. Details of the assay type and performance are specified in Additional file 1: Table S1.

### Covariates

All covariates were measured at Exam 1, which will serve as baseline for the HGF analyses, and Exam 2, which will serve as baseline for all other analyses. Demographics, education, smoking history, current alcohol use, and medication use were collected using self-administered and interviewer-administered questionnaires. In addition to the questionnaire, participants were asked to bring all medications to exams. Body mass index (BMI) was calculated as weight divided by height squared (kg/m^2^). Resting blood pressure was measured three times in the seated position using an automated oscillometric sphygmomanometer (Dinamap). The average of the last 2 readings was used in the analyses. Hypertension was defined as systolic blood pressure ≥ 140 mmHg, diastolic blood pressure (DBP) ≥ 90 mmHg, or taking antihypertensive medication. Serum glucose was assayed by a glucose oxidase method on the Vitros analyzer (Johnson & Johnson Clinical Diagnostic, Rochester, NY). Diabetes mellitus was defined as use of insulin or other diabetes mellitus medication, self-reported physician diagnosis, or fasting glucose ≥ 126 mg/dL. Glomerular filtration rate (GFR) was estimated using the Chronic Kidney Disease Epidemiology Collaboration formula [12]. Total cholesterol was measured in EDTA plasma using a cholesterol oxidase method (Roche Diagnostics, Indianapolis, IN) on a Roche COBAS FARA centrifugal analyzer. High-density lipoprotein (HDL) cholesterol was measured in EDTA plasma by the cholesterol oxidase method after precipitation of non-high-density lipoprotein cholesterol with magnesium/dextran (Roche Diagnostics, Indianapolis, IN). Heart failure (HF) was classified as definite or probable. Definite or probable HF both required HF symptoms such as shortness of breath or edema. Definite HF required one or more other criteria, such as pulmonary edema/congestion by chest X-ray; dilated ventricle or poor LV function; or evidence of left ventricular diastolic dysfunction. If definite HF criteria were not available, probable HF was defined as HF diagnosed by a physician and medical treatment for HF. Participants not meeting any criteria, including only a physician diagnosis of HF without any other evidence, were considered as having no HF.

### Atrial fibrillation ascertainment

Each participant was contacted at intervals of 9 to 12 months during follow-up to gather information on recent lifestyle changes and new CVD conditions, treatments, hospitalizations and procedures [11]. AF was identified using hospital discharge diagnoses, MESA study electrocardiograms, death certificates, or for those enrolled in fee-for-service Medicare, from inpatient and outpatient Medicare claims data. AF was defined by International Classification of Disease-9 (ICD-9) codes for AF or atrial flutter (ICD-9 codes 427.31 and 427.32, respectively). Incident AF was defined as the first occurrence of an AF diagnosis from baseline through the end of follow-up on December 31, 2014. A first diagnosis of AF made during the same hospitalization as cardiac surgery was not counted as incident AF, but a subsequent AF diagnosis in the same person, not associated with cardiac surgery, was considered their first AF episode. Follow-up for all analyses was calculated as the time elapsed from the baseline examination to whichever came first: first AF event, loss to follow-up, death, or December 31, 2014.

### Statistical methods

Bonferroni correction was used to account for multiple comparisons and a *P* value of < 0.0033 was considered statistically significant (0.05/15 proteins = 0.0033) for all analyses. Baseline characteristics of participants are presented by race/ethnicity and compared using the chi-squared test for categorical variables and the Kruskal–Wallis test for continuous variables. The association of protein levels with incident AF was assessed using Cox proportional hazards regression. For analyses of proteins measured at Exam 2, subjects with incident AF between Exams 1 and 2 were excluded and time was indexed to the Exam 2 visit date. We adjusted for known risk factors for CVD, and for atrial fibrillation specifically. Model 1 was adjusted for age, sex, and race/ethnicity. Model 2 was comprised of Model 1 covariates and height, BMI, systolic blood pressure, antihypertensive use, diabetes mellitus, smoking status, alcohol use, and total and HDL cholesterol. As a sensitivity analysis, heart failure was additionally added to Model 2 as a time-dependent variable as events occurred during follow-up to assess whether it was a plausible mediator in the association between proteins and incident AF. For all analyses, race/ethnicity-specific models were fit in addition to models pooling race/ethnicity that included race/ethnicity as a covariate. Additionally, we tested for multiplicative interactions of proteins with race/ethnicity and age by including cross-product terms in the pooled statistical model. Race/ethnicity was defined as White, Chinese, Black, and Hispanic. Due to the limited missing data at baseline and Exam 2 for the covariates of interest, imputation was not performed.

## Results

Table 1 summarizes the characteristics of MESA participants at Exam 2 that had plasma protein measurements (n = 2504; mean age 63; 47% male). The characteristics of the Exam 1 participants are summarized in Additional file 1: Table S2. The distributions of the proteins are summarized in Additional file 1: Figure S2. All of the protein levels varied by race/ethnicity, except for MMP-1 (Table 2).

**Table 1:** Exam 2 characteristics by race/ethnicity

Characteristics	Pooled sample n = 2504	African-American n = 607	Chinese-American n = 631	Hispanic-American n = 624	Non-Hispanic White-American n = 642
Age, years ± SD	63 ± 10	63 ± 10	63 ± 10	63 ± 10	63 ± 10
Male, %	47	46	49	46	48
Body mass index, kg/m^2^ ± SD	28 ± 6	30 ± 6	24 ± 3	30 ± 5	28 ± 5
Height, cm ± SD	165 ± 10	168 ± 10	162 ± 8	162 ± 9	169 ± 10
Systolic blood pressure, mmHg ± SD	124 ± 21	130 ± 22	121 ± 20	125 ± 21	122 ± 19
Antihypertensive medication use	39	52	33	36	37
Diabetes, % yes	16	20	14	20	9
Smoking status, % yes					
Never	51	42	73	50	38
Former	38	42	22	39	48
Current	12	17	5	11	14
Current use of alcohol, % yes	48	46	31	44	69
Total Cholesterol, mg/dL ± SD	193 ± 35	190 ± 37	190 ± 32	195 ± 35	194 ± 37
HDL cholesterol, mg/dL ± SD	51 ± 15	54 ± 16	50 ± 13	48 ± 13	54 ± 17
Heart Failure, %	3.7	3.6	2.9	4.5	3.7

*HDL* High density lipoprotein, *SD* standard deviation

**Table 2:** Unadjusted mean ± SD; median of protein levels by ethnicity/race at Exams 1 (2000–2002) and 2 (2002–2004), Multi-Ethnic Study of Atherosclerosis

Exam 1 protein	Pooled Sample (n = 6669)		African-American (n = 1841)		Chinese-American (n = 793)		Hispanic-American (n = 1469)		Non-Hispanic White American (n = 2566)		*P* value
	n	mean ± sd; median	n	mean ± sd; median	n	mean ± sd; median	n	mean ± sd; median	n	mean ± sd; median	
HGF, pg/mL	6669	937 ± 258; 903	1841	933 ± 249; 895	793	839 ± 216; 811	1469	1035 ± 268; 994	2566	915 ± 254; 884	< 0.001

Exam 2 proteins		(n = 2504)		(n = 607)		(n = 631)		(n = 624)		(n = 642)	*P* value
MMP-1, ng/mL	2349	5.4 ± 3.7; 4.4	559	5.7 ± 4.1; 4.6	593	5.4 ± 3.5; 4.5	591	5.2 ± 3.7; 4.2	606	5.3 ± 3.5; 4.4	0.23
MMP-2, ng/mL	2366	195 ± 31; 193	565	200 ± 33; 196	593	189 ± 28; 186	597	197 ± 31; 195	611	196 ± 30; 194	< 0.001
TIMP-2, ng/mL	2361	81 ± 12; 79	565	82 ± 13; 81	593	77 ± 11; 76	595	82 ± 11; 81	608	83 ± 12; 81	< 0.001
ICAM-1, ng/mL	2343	253 ± 87; 234	560	248 ± 86; 228	593	227 ± 75; 214	588	283 ± 98; 260	602	252 ± 78; 239	< 0.001
VCAM-1, ng/mL	2355	728 ± 203; 696	565	657 ± 193; 627	590	734 ± 203; 700	593	743 ± 199; 703	607	775 ± 200; 748	< 0.001
L-selectin, ng/mL	2366	889 ± 193; 874	569	868 ± 193; 854	592	830 ± 165; 820	597	901 ± 189; 891	608	953 ± 202; 940	< 0.001
RANTES, pg/mL	2473	4057 ± 3250; 3092	596	3987 ± 3611; 2844	629	4337 ± 3061; 3565	615	3971 ± 3273; 2933	633	3927 ± 3036; 3021	< 0.001
E-cadherin, ng/mL	2352	222 ± 58; 212	565	207 ± 53; 197	590	225 ± 57; 217	589	241 ± 60; 231	608	214 ± 54; 205	< 0.001
SLPI, pg/mL	2360	46,175 ± 10,505; 44,616	566	47,333 ± 11,654; 45,414	591	44,895 ± 10,153; 43,202	593	47,124 ± 9984; 45,897	610	45,420 ± 10,021; 44,518	< 0.001
IL-2 sR, pg/mL	2354	711 ± 301; 643	566	637 ± 258; 584	592	590 ± 227; 552	590	772 ± 289; 722	606	838 ± 344; 747	< 0.001
CCL-21, pg/mL	2361	803 ± 259; 759	566	797 ± 264; 750	592	772 ± 250; 739	595	807 ± 257; 775	608	833 ± 261; 788	< 0.001
SDF-1α, pg/mL	2497	1940 ± 441; 1908	605	1933 ± 462; 1889	629	1994 ± 429; 1962	622	1933 ± 436; 1895	641	1900 ± 433; 1873	0.001
P-selectin, ng/mL	2501	29 ± 10; 28	607	28 ± 10; 27	631	28 ± 9; 27	623	32 ± 10; 31	640	29 ± 11; 27	< 0.001
TGF-β1, pg/mL	2482	3880 ± 2084; 3434	600	3777 ± 2191; 3268	629	4361 ± 2164; 4011	618	3762 ± 2015; 3302	635	3616 ± 1882; 3184	< 0.001

*CCL-21* Chemokine ligand 21, *HGF* hepatocyte growth factor, *ICAM-1* intercellular adhesion molecule 1, *IL-2 sR* interleukin 2 soluble receptor, *MMP-1* matrix metalloproteinase 1, *MMP-2* matrix metalloproteinase 2, *RANTES* regulated on activation normal T cell expressed and secreted, *SLPI* secretory leukocyte protease inhibitor, *SD* standard deviation, *SDF-1α* stromal-derived factor 1a, *TGF-β1* transforming growth factor b1, *TIMP-2* tissue inhibitor of metalloproteinase 2, *VCAM-1* vascular cell adhesion molecule 1

During the 25,413 person-years of follow up from Exam 2 (average: 10.2 years), 253 incident cases of AF were identified (44 African Americans, 74 Chinese Americans, 57 Hispanic Americans, and 78 non-Hispanic white Americans). After adjustment for age, sex, and race/ethnicity, MMP-2, TIMP-2, VCAM-1, HGF, SLPI, and IL-2 sR were associated with a higher risk of AF with each standard deviation increase in protein level (*P* < 0.0033; Table 3).

**Table 3:** Adjusted hazard ratios for incident atrial fibrillation per one standard deviation increase in adhesion pathway protein

	Pooled sample				
	Model 1^a^		Model 2^b^		
	Hazard ratio (95% CI)	*P* value^c^	Hazard ratio (95% CI)	*P* value^c^	Race/Ethnicity interaction *P* value^c,d^
*Exam 1*					
HGF^e^	1.18 (1.10 -1.26)	< 0.0001	1.10 (1.02–1.18)	0.009	0.28
*Exam 2*					
MMP-1	1.02 (0.91–1.15)	0.72	1.02 (0.90–1.15)	0.76	0.36
MMP-2	1.25 (1.10–1.42)	< 0.001	1.27 (1.11–1.45)	< 0.001	0.14
TIMP-2	1.27 (1.11–1.44)	< 0.001	1.28 (1.12–1.46)	< 0.001	0.89
ICAM-1	1.11 (0.97–1.28)	0.13	1.09 (0.95–1.26)	0.23	0.51
VCAM-1	1.32 (1.17–1.50)	< 0.001	1.32 (1.16–1.5)	< 0.001	0.69
P-selectin	1.09 (0.86–1.40)	0.48	1.06 (0.83–1.37)	0.63	0.18
L-selectin	1.01 (0.88–1.15)	0.93	1.04 (0.90–1.20)	0.61	0.92
RANTES	1.02 (0.90–1.14)	0.8	1.03 (0.91–1.16)	0.68	0.80
E-cadherin	1.01 (0.88–1.15)	0.94	0.99 (0.85–1.14)	0.86	0.59
TGF-β1	1.00 (0.89–1.13)	0.97	0.99 (0.88–1.13)	0.97	0.79
CCL-21	1.20 (1.05–1.37)	0.008	1.17 (1.02–1.35)	0.025	0.76
SDF-1α	1.14 (1.00–1.30)	0.046	1.11 (0.97–1.27)	0.12	0.83
SLPI	1.22 (1.08–1.38)	0.001	1.22 (1.07–1.38)	0.003	0.02
IL-2 sR	1.22 (1.08–1.37)	0.001	1.20 (1.06–1.36)	0.004	0.22

*CCL-21* Chemokine ligand 21, *HGF* hepatocyte growth factor, *ICAM-1* intercellular adhesion molecule 1, *IL-2 sR* interleukin 2 soluble receptor, *MMP-1* matrix metalloproteinase 1, *MMP-2* matrix metalloproteinase 2, *RANTES* regulated on activation normal T cell expressed and secreted, *SDF-1α* stromal-derived factor 1α, *SLPI* secretory leukocyte protease inhibitor, *TGF-β1* transforming growth factor β1, *TIMP-2* tissue inhibitor of metalloproteinase 2, *VCAM-1* vascular cell adhesion molecule 1

^a^Adjusted for age, sex, and race/ethnicity. Proteins were not transformed for the analyses. Hazard ratios presented are for a 1 standard deviation unit change for each protein

^b^Adjusted for model 1 + height, body mass index, systolic blood pressure, antihypertension therapy, diabetes status, current smoker, current alcohol use, and total and HDL cholesterol

^c^Threshold for statistical significance *P* < 0.0033 (0.05/15)

^d^The race/ethnicity interaction term is not included in the final model. Stratified results are presented in Additional file 1: Table S3

^e^There were 74,960 person-years of follow up for the Exam 1 HGF analysis (average: 11.2 years), 851 incident cases of AF were identified (186 African Americans, 105 Chinese Americans, 151 Hispanic Americans, and 409 non-Hispanic white Americans)

Further adjustment for height, body mass index, systolic blood pressure, antihypertension therapy, diabetes status, current smoker, current alcohol use, and total and HDL cholesterol did not appreciably change point estimates for MMP-2, TIMP-2, VCAM-1, or SLPI. In contrast, full adjustment attenuated the association of HGF with AF (HR 1.10; 95% CI 1.02‒1.18) and IL-2 sR (HR 1.20; 95% CI 1.06‒1.36). Further adjustment for heart failure did not materially change any of the results. The association between proteins and AF did not differ by race/ethnicity (Additional file 1: Table S3) nor age (data not shown).

## Discussion

In a large, diverse population of over 2500 individuals (approximately 50% male), higher levels of circulating MMP-2, TIMP-2, VCAM-1, and SLPI were associated with a higher risk of AF, independent of traditional CVD risk factors. While there is growing evidence that adhesion pathway proteins may be associated with AF, prior studies have primarily focused on a limited number of proteins with small sample sizes and limited ethnic and racial diversity [6–10]. Thus, by studying a large number of proteins in a large, ethnic and racially diverse population, we were able to comprehensively evaluate the association between proteins in the cellular adhesion pathway and risk of AF. Our findings have important implications for preventing and improving care of patients at risk for AF.

We found a significant association between MMP-2 and risk of AF. MMP-2 is a gelatinase and in addition to breaking down extracellular matrix, MMP-2 is thought to play a role in proliferation, vascular remodeling, and oxidized LDL induced smooth muscle migration [13–15]. Xu et al. found that upregulated MMP-2 expression and downregulated TIMP-2 expression in the atrial tissue of AF patients was associated with the development of sustained AF [16]. Diao and colleagues also demonstrated that persistent AF patients had notably increased MMP-2 expression in left atrial tissue [10]. One prospective study found that increased circulating levels of MMP-2 were independent predictors of myocardial infarction, stroke, peripheral embolization, and death among patients with AF [17]. These associations give further insight on the role of MMP-2 in the mechanism of atrial remodeling and the development and perpetuation of AF.

There was an association between higher levels of TIMP-2 and increased risk of AF. TIMP-2 is a metallopeptidase inhibitor and directly suppresses the proliferation of endothelial cells through MMP-dependent mechanisms. In contrast to our findings, results from a meta-analysis found that lower circulating levels of TIMP-2 were associated with an increased risk of AF, albeit inclusion of different subtypes of AF was postulated as an explanation for the inconsistent findings with prior literature [18]. The higher circulating levels of TIMP-2 found in our study might be explained by its activation upon elevated levels of MMP-2, or by abnormal myocyte response in the remodeled atria [19].

Similar to previous studies, we found a positive association between VCAM-1 and risk of AF [8, 9, 20]. VCAM-1 is a member of the immunoglobulin family of adhesion proteins and in tandem with its receptor, VLA-4, mediates leukocyte-endothelial cell adhesion and signal transduction [21]. Of the two prospective cohort studies assessing the association of VCAM-1 with incident AF, one reported that baseline levels of VCAM-1 predicted post-operative AF after coronary artery bypass surgery and the other found that soluble levels of VCAM-1 had a positive, dose–response relationship with long-term risk of AF [8, 9]. Our study corroborates these findings and expands on the prior work by using a racially/ethnically diverse population.

SLPI was associated with AF risk. SLPI has anti-inflammatory actions regulating TGF-βand IL10 production and suppressing MMP production and activity [22–25]. Normally found in mucosal tissue, monocytes, and neutrophils, SLPI is expressed in epithelial cells during injury and is thought to prevent the excessive tissue destruction associated with delayed wound healing [26, 27]. To the best of our knowledge, no other studies have assessed the relationship between SLPI and AF.

After full adjustment for confounders, HGF and IL-2 sR were modestly associated with AF. IL-2 sR is the soluble form of the receptor for IL-2, a cytokine well-known for its role in T-lymphocyte function [28, 29]. The soluble receptor of IL-2 is secreted in response to inflammation [28, 29]. Evidence suggests that inflammation seems to trigger AF, although AF might help maintain an inflammatory environment as well [4]. HGF is produced in vascular cells, functions as a potent inducer of LFA-1, and reduces surface expression of L-selectin [30]. Higher circulating levels of HGF have been reported in patients with CVD [31–33]. Li et al. found that local HGF levels in samples from the right atrium of AF patients were reduced, confirming dysfunction in the local production of HGF. Systemic HGF levels have been found to increase via the endocrine pathway from sources like the lungs and liver as a result of vascular endothelial injury [34]. Katoh et al. reported higher circulating levels of HGF in patients with non-valvular AF [31]. The association we found between HGF and AF is consistent with this result.

### Clinical implications

In this study we found that several proteins in the cellular adhesion pathway, specifically MMP-2, TIMP-2, VCAM-1, and SLPI, are associated with increased risk of AF. These findings have important clinical implications. Our results indicate that strategies aimed at reducing cellular adhesion during the inflammatory response may prevent risk of AF. However, further investigation is needed to determine whether interventions targeting components of the adhesion pathway could eventually lead to new tools for defining AF risk or prevention of AF.

### Limitations

The present study had some limitations. First, incident AF is likely underestimated given that asymptotic AF may go undetected. As a result, we could be underestimating the association of proteins and AF due to misclassification or our results could be specific to symptomatic cases. We were underpowered to detect association differences across races/ethnicities. Thus, assessing the heterogeneity by race/ethnicity in larger populations is an area of future research. Finally, proteins were measured in the cohort at a single point in time and AF events could have occurred more than a decade after their measurement. However, circulating levels of these proteins have been shown to be relatively stable over time, indicating that a single measurement is a reasonable biomarker for an individual’s exposure to the proteins [35, 36].

## Conclusions

In conclusion, soluble levels of MMP-2, TIMP-2, VCAM-1, and SLPI were associated with an increased risk of AF in a diverse population from the United States. Our findings suggest that adhesion pathway proteins may be important risk predictors of AF.

## Supplementary Information

**Additional file 1:** **Table S1.** Assay characteristics by protein, Multi-Ethnic Study of Atherosclerosis, 2002-2004. **Table S2.** Exam 1 characteristics by race/ethnicity, Multi-Ethnic Study of Atherosclerosis, 2000-2002. **Table S3.** Adjusted hazard ratios for incident atrial fibrillation per one standard deviation increase in protein by ethnicity/race, Multi-Ethnic Study of Atherosclerosis, 2002-2014. **Figure S1.** Flow diagram of Exam 2 study participation. **Figure S2.** Distribution of proteins levels.

## Data Availability

The datasets analyzed during the current study may be requested from the MESA Coordinating Center.

## Abbreviations

AF: Atrial fibrillation
BMI: Body mass index
CCL-21: Chemokine ligand 21
CRP: C-reactive protein
CVD: Cardiovascular disease
DBP: Diastolic blood pressure
GFR: Glomerular filtration rate
HDL: High-density lipoprotein
HF: Heart failure
HGF: Hepatocyte growth factor
ICAM: Intercellular adhesion molecule 1
ICD-9: International Classification of Disease-9
IL-2 sR: Interleukin 2 soluble receptor
MMP-1: Matrix metalloproteinase 1
MMP-2: Matrix metalloproteinase 2
MESA: Multi-Ethnic Study of Atherosclerosis
RANTES: Regulated on activation, normal T cell expressed and secreted
SDF-1α: Stromal-derived factor 1α
TGF-β1: Transforming growth factor β1
SLPI: Secretory leukocyte protease inhibitor
TIMP-2: Tissue inhibitor of metalloproteinase 2
VCAM-1: Vascular cell adhesion molecule 1
