# Supplementary material for: Adhesion pathway proteins and risk of atrial fibrillation in the Multi-Ethnic Study of Atherosclerosis
Source: BMC Cardiovasc Disord. 2021 Sep 14;21:436. doi: 10.1186/s12872-021-02241-w (PMC8442417; doi:10.1186/s12872-021-02241-w)
Supplement: Supplementary file 1 — Additional file 1: Table S1. Assay characteristics by protein, Multi-Ethnic Study of Atherosclerosis, 2002-2004. Table S2. Exam 1 characteristics by race/ethnicity, Multi-Ethnic Study of Atherosclerosis, 2000-2002. Table S3. Adjusted hazard ratios for incident atrial fibrillation per one standard deviation increase in protein by ethnicity/race, Multi-Ethnic Study of Atherosclerosis, 2002-2014. Figure S1. Flow diagram of Exam 2 study participation. Figure S2. Distribution of proteins levels. [file 12872_2021_2241_MOESM1_ESM.docx]

**Supplementary Information**

**Adhesion pathway proteins and risk of atrial fibrillation in the Multi-Ethnic Study of Atherosclerosis**

**Table S1 Assay characteristics by protein, Multi-Ethnic Study of Atherosclerosis, 2002-2004**

**Table S2 Exam 1 characteristics by race/ethnicity, Multi-Ethnic Study of Atherosclerosis, 2000-2002**

**Table S3 Adjusted hazard ratios for incident atrial fibrillation per one standard deviation increase in protein by ethnicity/race, Multi-Ethnic Study of Atherosclerosis, 2002-2014**

**Figure S1 Flow diagram of Exam 2 study participation**

**Figure S2 Distribution of proteins levels**

| **Table S1 Assay characteristics by protein, Multi-Ethnic Study of Atherosclerosis, 2002-2004** | | | |
| --- | --- | --- | --- |
| Protein | ELISA | Minimum  detection level | Inter-assay coefficient  of variation (%) |
| MMP-1, ng/mL | Human Pro-MMP-1 Quantikine ELISA Kit (R&D Systems, Minneapolis, MN) | 0.021 ng/mL | 3.5% at a mean concentration of 6.3 ng/mL |
| MMP-2, ng/mL | Total MMP-2 Quantikine ELISA Kit (R&D Systems, Minneapolis, MN) | 0.047 ng/mL | 3.8% at a mean concentration of 186 ng/mL |
| TIMP-2, ng/mL | Human TIMP-2 Quantikine ELISA Kit (R&D Systems, Minneapolis, MN) | 0.011 ng/mL | 4.7% at a mean concentration of 73.3 ng/mL |
| ICAM-1, ng/mL | Human sICAM-1 Instant ELISA (Bender MedSystems GmbH, Vienna, Austria) | 2.17 ng/mL | 9.1% at a mean concentration of 261 ng/mL |
| VCAM-1, ng/mL | Human sVCAM-1/CD106 Quantikine ELISA Kit (R&D Systems, Minneapolis, MN) | 0.6 ng/mL | 3.6% at a mean concentration of 564 ng/mL |
| P-selectin, ng/mL | Human soluble P-selectin/CD62P Immunoassay Kit (R&D Systems, Minneapolis, MN) | 0.5 ng/mL | 6.7% at a mean concentration of 182.1 ng/mL |
| L-selectin, ng/mL | Human soluble L-selectin/CD62L Immunoassay Kit (R&D Systems, Minneapolis, MN) | 0.3 ng/mL | 6.7% at a mean concentration of 943 ng/mL |
| RANTES, pg/mL | Human CCL5/RANTES Quantikine ELISA Kit (R&D Systems, Minneapolis, MN) | 2.0 pg/mL | 10.0% at a mean concentration of 63,287 pg/mL |
| E-cadherin, ng/mL | Human E-cadherin Quantikine ELISA Kit (R&D Systems, Minneapolis, MN) | 0.039 ng/mL | 7.8% at a mean concentration of 197 ng/mL |
| TGF-β1, pg/mL | Human TGF-beta 1 Quantikine ELISA Kit (R&D Systems, Minneapolis, MN) | 4.61 pg/mL | 9.1% at a mean concentration of 32,223 pg/mL |
| ^a^HGF, pg/mL | Human HGF Quantikine ELISA Kit (R&D Systems, Minneapolis, MN) | 40 pg/mL | 10.4% at a mean concentration of 688 pg/mL |
| CCL-21, pg/mL | Human CCL21/6Ckine Quantikine ELISA Kit (R&D Systems, Minneapolis, MN) | 9.9 pg/mL | 5.6% at a mean concentration of 493 pg/mL |
| SDF-1α, pg/mL | Human CXCL12/SDF-1 alpha Quantikine ELISA Kit (R&D Systems, Minneapolis, MN) | 18 pg/mL | 11.40% at a mean concentration of 2,228 pg/mL |
| SLPI, pg/mL | Human SLPI Quantikine ELISA Kit (R&D Systems, Minneapolis, MN) | 25 pg/mL | 8.9% at a mean concentration of 36,888 pg/mL |
| IL-2 sR, pg/mL | Human IL-2 sR Quantikine ELISA Kit (R&D Systems, Minneapolis, MN) | 10 pg/mL | 7.7% at a mean concentration of 653 pg/mL |

CCL-21: chemokine ligand 21; HGF: hepatocyte growth factor; ICAM-1: intercellular adhesion molecule 1; IL-2 sR: interleukin 2 soluble receptor; MMP-1: matrix metalloproteinase 1; MMP-2: matrix metalloproteinase 2; RANTES, regulated on activation normal T cell expressed and secreted; SDF-1α: stromal-derived factor 1a; SLPI: secretory leukocyte protease inhibitor; TGF-β1: transforming growth factor β1; TIMP-2: tissue inhibitor of metalloproteinase 2; VCAM-1: vascular cell adhesion molecule 1.

^a^Measured at Exam 1 (2000-2002).

| **Table S2 Exam 1 characteristics by race/ethnicity, Multi-Ethnic Study of Atherosclerosis, 2000-2002** | | | | | |
| --- | --- | --- | --- | --- | --- |
| Characteristics (Means or Prevalences Unless Otherwise Stated) | Pooled Sample  (n=6669) | African-American (n=1841) | Chinese-American (n=793) | Hispanic-American  (n=1469) | Non-Hispanic  White-American  (n=2566) |
| Age, years ± SD | 62 ± 10 | 62 ± 10 | 62 ± 10 | 61 ± 10 | 62 ± 10 |
| Male, % | 47 | 45 | 49 | 48 | 48 |
| Body mass index, kg/m² ± SD | 28 ± 6 | 30 ± 6 | 24 ± 3 | 29 ± 5 | 28 ± 5 |
| Height, cm ± SD | 166 ± 10 | 168 ± 10 | 162 ± 9 | 162 ± 9 | 169 ± 10 |
| Systolic blood pressure, mmHg ± SD | 127 ± 21 | 132 ± 22 | 125 ± 22 | 127 ± 22 | 123 ± 20 |
| Antihypertensive medication use, % yes | 37 | 50 | 29 | 32 | 33 |
| Diabetes, % yes | 12 | 17 | 13 | 17 | 6 |
| Smoking status |  |  |  |  |  |
| Never smokers, % yes | 50 | 45 | 75 | 54 | 44.5 |
| Former smokers, % yes | 37 | 37 | 19 | 33 | 44.0 |
| Current smokers, % yes | 13 | 18 | 6 | 14 | 11.5 |
| Current use of alcohol, % yes | 56 | 50 | 32 | 48 | 72.0 |
| Total Cholesterol, mg/dL ± SD | 194 ± 36 | 190 ± 36 | 193 ± 32 | 198 ± 38 | 196 ± 35 |
| HDL cholesterol, mg/dL ± SD | 51 ± 15 | 52 ± 15 | 50 ± 13 | 48 ± 13 | 52.2 ± 15.6 |

HDL: high density lipoprotein; SD: standard deviation.

| **Table S3 Adjusted hazard ratios for incident atrial fibrillation per one standard deviation increase in protein by ethnicity/race,**  **Multi-Ethnic Study of Atherosclerosis, 2002-2014** | | | | | | | | | |  |
| --- | --- | --- | --- | --- | --- | --- | --- | --- | --- | --- |
|  | African-American | | Chinese-American | | Hispanic-American | | Non-Hispanic White American | |  | |
|  | Model 2^a^ | *P* value | Model 2^a^ | *P* value | Model 2^a^ | *P* value | Model 2^a^ | *P* value |  |  |
|  | Hazard Ratio  (95% CI) |  | Hazard Ratio  (95% CI) |  | Hazard Ratio  (95% CI) |  | Hazard Ratio  (95% CI) |  | Ethnicity/Race Interaction  *P* value | |
| Adhesion protein | |  |  |  |  |  |  |  |  |  |
| MMP-1 | 0.78  (0.56-1.07) | 0.13 | 0.99  (0.77-1.28) | 0.96 | 1.08  (0.83-1.42) | 0.56 | 1.14  (0.91-1.42) | 0.26 | 0.36 |  |
| MMP-2 | 0.99  (0.70-1.39) | 0.95 | 1.24  (0.97-1.59) | 0.09 | 1.18  (0.89-1.57) | 0.25 | 1.57  (1.22-2.03) | < 0.001 | 0.14 |  |
| TIMP-2 | 1.26  (0.92-1.74) | 0.15 | 1.22  (0.93-1.58) | 0.15 | 1.24  (0.91-1.69) | 0.17 | 1.37  (1.09-1.74) | 0.01 | 0.89 |  |
| ICAM-1 | 1.04  (0.73-1.50) | 0.83 | 1.02  (0.76-1.35) | 0.92 | 1.12  (0.88-1.42) | 0.37 | 1.0  (0.73-1.38) | 0.98 | 0.51 |  |
| VCAM-1 | 1.30  (0.94-1.80) | 0.11 | 1.36  (1.08-1.70) | 0.01 | 1.18  (0.88-1.58) | 0.26 | 1.29  (1.01-1.64) | 0.04 | 0.69 |  |
| P-selectin | 1.25  (0.73-2.15) | 0.41 | 0.66  (0.31-1.39) | 0.27 | 1.12  (0.61-2.06) | 0.71 | 1.10  (0.77-1.59) | 0.60 | 0.18 |  |
| L-selectin | 1.11  (0.81-1.53) | 0.52 | 0.95  (0.72-1.24) | 0.70 | 0.95  (0.70-1.29) | 0.75 | 1.08  (0.83-1.41) | 0.55 | 0.92 |  |
| RANTES | 1.07  (0.83-1.38) | 0.61 | 0.97  (0.76-1.23) | 0.77 | 1.08  (0.83-1.40) | 0.58 | 1.07  (0.83-1.38) | 0.60 | 0.80 |  |
| E-cadherin | 1.14  (0.82-1.58) | 0.44 | 1.0  (0.75-1.32) | 0.97 | 0.90  (0.66-1.22) | 0.49 | 0.94  (0.71-1.25) | 0.68 | 0.59 |  |
| TGF-β1 | 1.10  (0.83-1.44) | 0.52 | 1.01  (0.81-1.26) | 0.94 | 1.06  (0.81-1.38) | 0.69 | 0.95  (0.72-1.26) | 0.74 | 0.79 |  |
| ^b^HGF | 1.16  (1.00-1.35) | 0.05 | 1.16  (0.92-1.46) | 0.20 | 1.18  (1.01-1.38) | 0.04 | 1.04  (0.93-1.15) | 0.54 | 0.28 |  |
| CCL-21 | 1.36  (0.99-1.85) | 0.05 | 1.02  (0.77-1.33) | 0.91 | 1.06  (0.79-1.41) | 0.72 | 1.06  (0.80-1.40) | 0.70 | 0.76 |  |

| SDF-1α | 0.98  (0.74-1.31) | 0.91 | 1.07  (0.84-1.37) | 0.59 | 1.21  (0.89-1.64) | 0.22 | 1.22  (0.95-1.56) | 0.12 | 0.83 |
| --- | --- | --- | --- | --- | --- | --- | --- | --- | --- |
| SLPI | 1.09  (0.82-1.44) | 0.55 | 1.57  (1.26-1.96) | < 0.001 | 0.99  (0.74-1.33) | 0.95 | 1.02  (0.78-1.34) | 0.88 | 0.02 |
| IL-2 sR | 1.54  (1.11-2.14) | 0.01 | 1.38  (1.05-1.81) | 0.02 | 1.0  (0.75-1.33) | 0.98 | 1.12  (0.91-1.37) | 0.28 | 0.22 |

CCL-21: chemokine ligand 21; HGF: hepatocyte growth factor; ICAM-1: intercellular adhesion molecule 1; IL-2 sR: interleukin 2 soluble receptor; MMP-1: matrix metalloproteinase 1; MMP-2: matrix metalloproteinase 2; RANTES: regulated on activation normal T cell expressed and secreted; SDF-1α: stromal-derived factor 1a; SLPI: secretory leukocyte protease inhibitor; TGF-β1: transforming growth factor β1; TIMP-2: tissue inhibitor of metalloproteinase 2; VCAM-1: vascular cell adhesion molecule 1.

^a^Adjusted for model 1 + height, body mass index, systolic blood pressure, antihypertensive medication use, diabetes status, current smoker, current alcohol use, and total and HDL cholesterol.

^b^Measured at Exam 1 (2000-2002).

**Figure S1 Flow diagram of Exam 2 study participation**


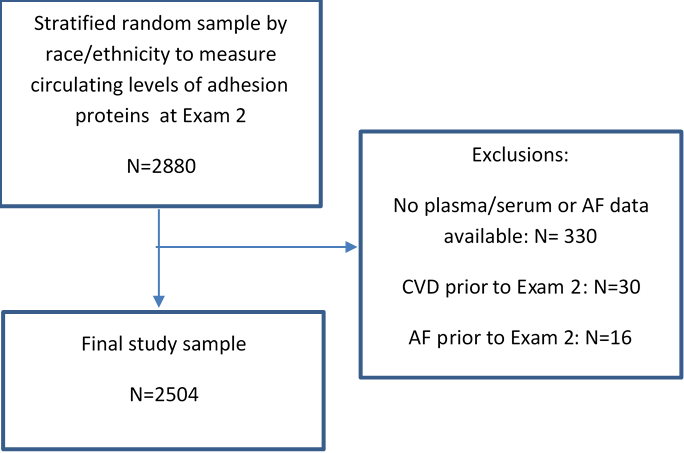


**Figure S2 Distribution of proteins levels**
